# Evaluation of a collaborative care system in primary care in the implementation of ambulatory follow-up in mental health: A study protocol

**DOI:** 10.1371/journal.pone.0343295

**Published:** 2026-02-25

**Authors:** Manuel Dias Alves, Asmaa Jobic, Natacha Rosso, Manon Abello, Aurélie Autret, Jean Vion-Dury, Raoul Belzeaux, Fabien Korrichi

**Affiliations:** 1 Centre Hospitalier Intercommunal de Toulon La Seyne-sur-Mer, Toulon, France; 2 CEReSS-Health Service Research and Quality of Life Centre, School of Medicine - La Timone Medical, Aix-Marseille University, Marseille, France; 3 Délégation à la Recherche Clinique et à l’Innovation, Centre Hospitalier Intercommunal de Toulon La Seyne-sur-Mer, Toulon, France; 4 Univ. Bordeaux, CNRS, SANPSY, UMR 6033, Bordeaux, France; 5 IGF, Université de Montpellier, CNRS, INSERM, Montpellier, France; 6 Department of Psychiatry, CHU Montpellier, Montpellier, France; PLOS: Public Library of Science, UNITED STATES OF AMERICA

## Abstract

Access to secondary psychiatric consultation is often challenging for general practitioners (GPs), potentially resulting in treatment delays or increased reliance on emergency services. The *med@psy* system is a collaborative care platform that enables GPs to schedule consultation appointments with psychiatrists within 48 hours. This study aims to compare the implementation of outpatient follow-up between two care pathways: attendance at a psychiatric emergency department for a consultation followed by discharge home and psychiatric consultation referred by the patient’s GP via the *med@psy* collaborative care system. The study consists of two prospective, multicenter, comparative and nonrandomized cohorts. Patients will be teleconsulted at one month and again at three months to assess whether they have received appropriate outpatient follow-up. This is a real-life study involving GPs and psychiatrists within a collaborative care model. The strengths and limitations of this study are discussed below. The results of this study will help gather valuable data on these two care pathways and will serve as a basis for improving and optimizing patient management strategies in outpatient mental health care.

Trial registration

ClinicalTrials.gov NCT06581874

## Introduction

Access to care and unscheduled care are major public health concerns. The problem is even more significant in psychiatry, where the COVID-19 pandemic has further increased the demand for care in an already overburdened system. According to the French COVID-19REV survey (wave 37) conducted in September 2023, 23% of respondents reported signs of anxiety, and 16% reported signs of depression [1]. A significant proportion of these situations (25%), known as mild or moderate disorders, can be managed in primary care if general practitioners (GPs) have access to psychiatric referrals [2].

General practitioners (GPs) are the primary point of contact for mental health services in France and numerous countries [3]. They play a strategic role in the coordination and management of psychiatric disorders [4,5]. The coordinated care pathway is separated into two referral levels: primary care, centered around GPs, who are responsible for patient referral, and secondary care, which is provided by specialists and health establishments [6] and is intended for cases requiring more complex interventions. The deployment of earlier and more graduated care has the advantage of reducing the intensity of psychiatric disorders and their repercussions [7]. Initial consultation with psychiatrists should take place as early as possible. However, this hierarchical model has limitations. Access to second-line psychiatric care can be difficult, leading to treatment delays or referrals to an unsuitable care pathway. Psychiatric emergency departments are becoming the gateway to psychiatric care. This trend is accompanied by an increase in emergency visits, including a growing number of “inappropriate or avoidable visits” [8].

Cooperation between GPs and psychiatrists is therefore essential. The collaborative care model facilitates this cooperation. Collaborative mental health care can be defined as *the process in which primary care and mental health professionals share resources, expertise, knowledge and decision-making to ensure that primary care populations receive care that is person-centered, effective, efficient and delivered by the appropriate professional* [9]. Recommendations for the mental health field have already been formalized [10].

**Fig 1:**
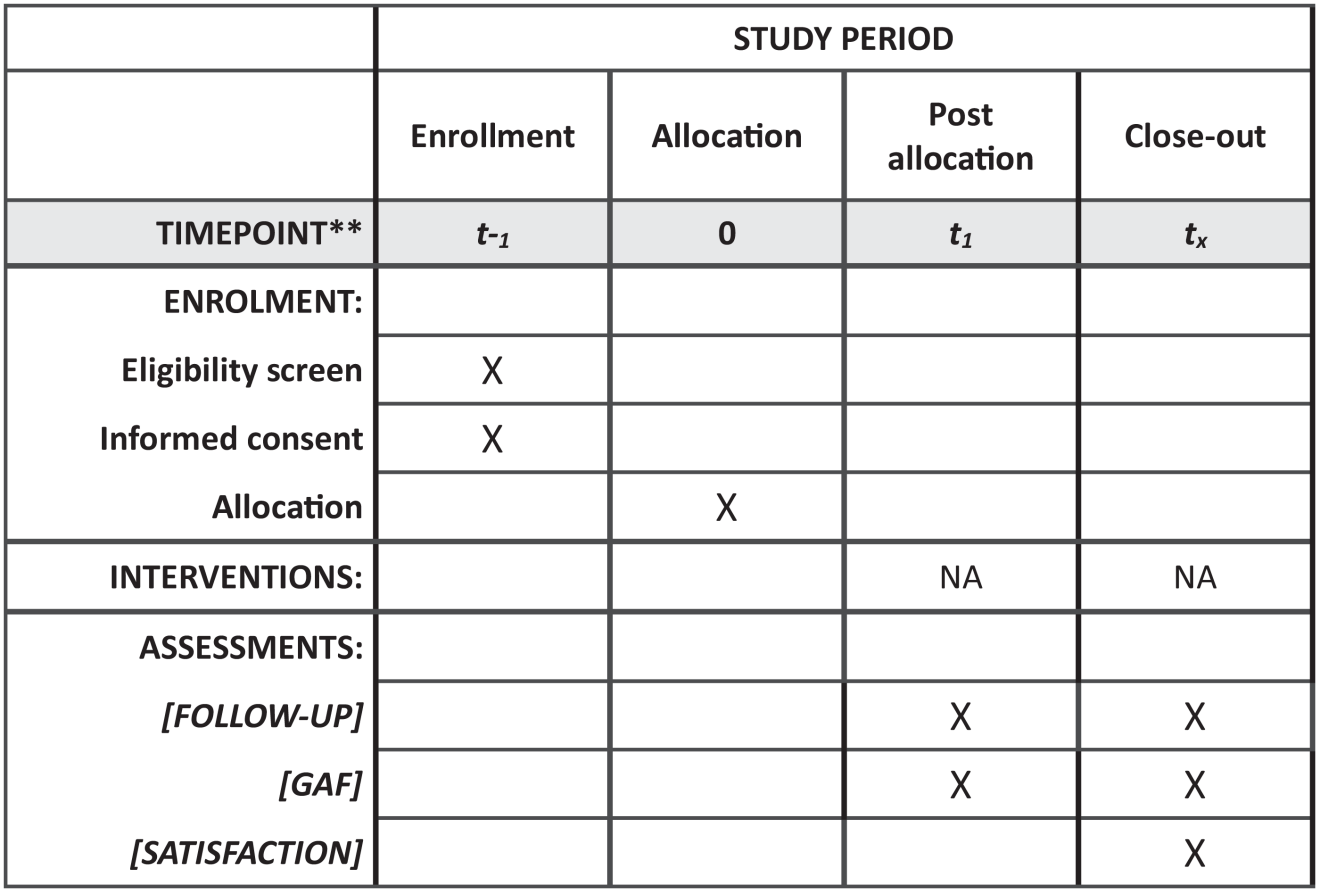
Description of the *med@psy* system user circuit.

Analysis of the issues described above led to the development of the *med@psy* system, supported by the digital platform *med@med.* This is an innovative collaborative care system in the mental health care pathway. The *med@psy* system combines human (telephone support) and digital logistical support to help GPs address the difficulties and isolation they often face when dealing with psychiatric cases in daily practice. GPs use this system to guide their patients toward the most appropriate care pathway, with step-by-step assistance provided by a medical decision support system. Fig 1 shows how the *med@psy* system works. The *med@psy* system encourages organization and collaboration between GPs and psychiatrists. It supports the implementation of primary mental health care by facilitating timely access to specialist input. This allows GPs to obtain real-time secondary psychiatric consultations within 48 hours through a shared care model, enhancing coordinated patient management and continuity of care.

Three main categories of psychiatric clinical conditions can lead to consultations at the emergency department: “pure” psychiatric emergencies (acute decompensation of a characterized psychiatric condition), “mixed” emergencies (involving both psychiatric and nonpsychiatric symptoms) and “transient acute states” (intense emotional reactions) [11]. The *med@psy* system mainly addresses acute transient states in which there is no immediate risk of action, such as suicide attempts. It can also address prodromal states or symptomatic exacerbations in patients with an established psychiatric disorder who are being followed by a general practitioner.

The system has been specifically set up at Métropole Toulon Provence Méditerranée. In 2018, this urban area had a population of 438,985 for 366.41 km^2^, with 83 psychiatrists and 436 GPs. Within this territory, between 1 July 2021 and 30 June 2022, 1460 psychiatric consultations were conducted in the psychiatric emergency unit of the Toulon – La Seyne-sur-Mer Intercommunal Hospital Center (CTS), with patients discharged home without hospitalization. Among these patients, 80% required outpatient follow-up (n = 1168), and it was estimated that only 10% of patients received follow-up one month later. The *med@psy* system differs from existing systems in other French regions because it is based on a single-entry point through GPs. It takes advantage of existing networks (both hospital and private practices) by stimulating them to optimize the link between GPs and psychiatrists. Its advantages include ease of use, digital efficiency and support for medical decision-making, allowing a tailored response to the patient’s need to be proposed in less than five minutes. The system promotes patient involvement and ensures that the time between the GP’s request and the patient’s appointment validation is under two hours. The system facilitates the scheduling of appointments within 48 hours of referral. It operates through a stepped referral process: initially, a personalized referral to a specific psychiatrist is made; if the psychiatrist is unavailable, the referral is voluntarily extended to the entire network, and in cases of no response, it is automatically redirected to other psychiatric network resources to ensure timely care. Furthermore, the coordination efforts of both the general practitioner and the psychiatrist are acknowledged through increased reimbursement for consultations.

### Aim

We hypothesize that the *med@psy* system, which enables patients to obtain a psychiatric consultation within 48 hours of referral from a general practitioner, facilitates the rapid implementation of outpatient follow-up. This study aims to compare the implementation of outpatient follow-up between two care pathways: one visit to the psychiatric emergency department with the patient discharged home without hospitalization and one psychiatrist consultation via the *med@psy* system following referral by the patient’s GP.

## Materials and methods

### Study design

This study is designed as a prospective, multicenter, nonrandomized comparative cohort study involving two distinct groups: Group 1 and Group 2. Group 1 consisted of patients who presented to the emergency department of the Toulon-La Seyne-sur-Mer Intercommunal Hospital Center (CTS) regardless of whether they were referred by their GP. Group 2 consisted of patients referred by their GP for a psychiatric consultation through the *med@psy* system. Patients will be selected and included from two investigating centers: the *med@psy* system, which is attached to psychiatric practice, and the CTS Emergency Department.

### Procedure

All patients meeting the eligibility criteria will be identified following their consultation, either at the CTS psychiatric emergency department with an outpatient discharge (Group 1) or following referral by their GP for a psychiatric consultation scheduled within 48 hours *via* the *med@psy* system (Group 2).

**Fig 2:**
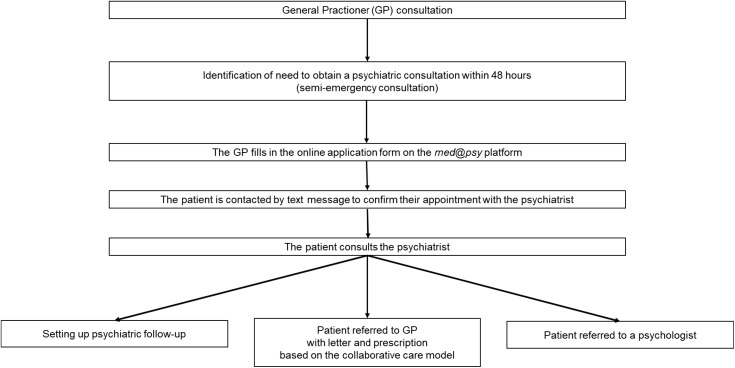
Schedule of enrollment, interventions, and assessments.

**Fig 3:**
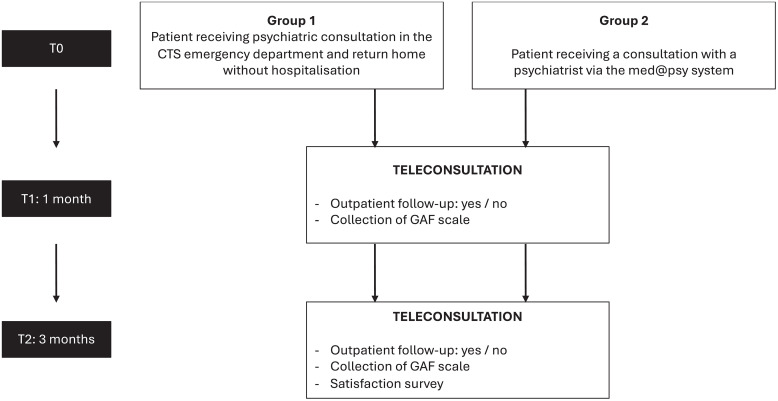
Description of the study process. (GAF: Global Assessment of Functioning).

Prior to any examination or research-related procedure, the investigator will obtain the patient’s non-opposition of the person undergoing the research. The identified patients will be contacted by telephone by the investigator or a member of the team to be informed about the research. The information will be given both orally and in writing, using a non-opposition information note. It will be written in clear language that the patient can fully understand. Patients will be given sufficient time to consider their participation. The enrollment, intervention, and assessment schedules are presented in Fig 2 and the study flowchart is shown in Fig 3.

For Group 1, after a clinical assessment by a psychiatrist in the emergency department that does not lead to hospitalization, the patient will be given a psychiatric consultation and discharged. The patients were contacted within one week of their visit to the emergency department to be informed about the research. If the patient does not object to participation, they will be contacted at one month and three months post-visit by a psychologist, who will administer the Global Assessment of Functioning (GAF) scale and collect information regarding any follow-up with a psychologist, psychiatrist or general practitioner.

For Group 2, patients will be seen within 48 hours of consulting their GP by a psychiatrist taking part in the *med@psy* system. As part of the research, patients will be contacted within a week of this consultation to be informed about the research. If the patient does not object to participation, they will be contacted at one month and three months after their consultation via the application by the study psychologist, who will administer the GAF scale and collect information regarding any follow-up with a psychologist, psychiatrist or general practitioner.

### Participants sample size

The study will include all patients meeting the inclusion and exclusion criteria over 12 months. Between 1 July 2021 and 30 June 2022, 1460 psychiatric consultations were carried out in the CTS psychiatric emergency department, with patients discharged home without hospitalization. If 80% of these patients are estimated to require outpatient follow-up, this corresponds to 1168 patients.

Based on preliminary estimates, 10% of patients seen in the emergency departments (Group 1) are expected to have outpatient follow-up set up at one month, compared with 20% of patients seen *via* the *med@psy* system (Group 2). Considering the null hypothesis as the equality of two proportions, with a first order risk (alpha) of 5%, the simple size calculation using the SAS (*Statistical Analysis Software*) power procedure estimates that a total of 434 patients are required (217 per group) to detect a statistically significant difference with a power of at least 90%. When a 10% drop-out rate is allowed, a minimum of 239 patients per group should be included, which seems plausible over a 12-month inclusion period.

### Inclusion and exclusion criteria

The inclusion criteria were as follows: (1) patients aged 18 years or older; (2) patients presenting to the CTS psychiatric emergency department, with or without referral, who received a psychiatric consultation and were discharged home without hospitalization after clinical evaluation (Group 1) or who were referred for psychiatric consultation *via* the *med@psy* system (Group 2); and (3) patients with indications for outpatient follow-up. (4) Patients with valid social security entitlements.

The exclusion criteria were as follows: (1) patient opposition to participate in the research and (2) patient was currently under psychiatrist follow-up. Psychiatric follow-up is defined as at least one consultation with a psychiatrist within the past three months, excluding consultations for unscheduled or emergency care; (3) patients referred for a suicide attempt, violent or nonviolent, serious or nonserious; (4) patients with no declared GP; (5) patients already included in the study; (6) patients who do not speak French; (7) patients under judicial protection or safeguard of justice; (8) patients under obligation care, under care order or deprived of liberty; and (9) any other reason that, in the investigator’s opinion, could interfere with the evaluation of the study objectives.

### Outcomes

The main objective of this study was to compare the proportion of patients with a psychiatric disorder who received outpatient follow-up one month after a visit to a psychiatric emergency department without hospitalization (Group 1), *versus* one month after a psychiatrist consultation conducted within 48 hours *via* the *med@psy* *medical* system (Group 2). The primary outcome is the presence or absence of outpatient follow-up, which is defined as at least one consultation with a psychiatrist (excluding psychiatric consultations at the emergency department or psychiatric consultations *via* the *med@psy* system), a psychologist or a general practitioner. Only GP consultations specifically related to psychiatric reasons were considered. This information will be collected during the teleconsultation visit with the psychiatrist at one month. The one-month follow-up period begins from the date of the psychiatric consultation *via* *either* the *med@psy* system or the CTS emergency department. This timeframe was chosen because it is assumed that there is a delay of approximately three weeks for the implementation of outpatient follow-up for Group 1 patients, which corresponds to the average wait time for the first consultation at the medical-psychological centers in the Métropole Toulon Provence Méditerranée area.

The primary secondary objective was to compare the proportion of patients with a psychiatric disorder who received outpatient follow-up three months after a visit to a psychiatric emergency department without hospitalization (Group 1) *versus* three months after a consultation with a psychiatrist within 48 hours *via* the *med@psy* system (Group 2). As with the primary outcome, outpatient follow-up is defined by at least one consultation with a psychiatrist (excluding emergency department or *med@psy* consultations), a psychologist, or a GP for psychiatric reasons.

The second secondary objective was to compare the overall psychological, social and professional functioning of patients in the two groups via the Global Assessment of Functioning (GAF) scale. The assessment will be conducted by a psychologist during teleconsultations carried out at one and three months. The GAF scale, a component of the Diagnostic and Statistical Manual of Mental Disorders (DSM-IV), provides a global score to subjectively rate the social, occupational, and psychological functioning of an individual. Scores range from 100 (extremely high functioning) to 1 (severely impaired). It incorporates the evaluation of psychological, social, and occupational impairments and considers any physical or social problems that may influence treatment or the severity of the psychiatric condition.

The third secondary objective is to evaluate the adherence of identified medical partners to the use of the *medication* system on the basis of data collected directly from the platform. Adherence will be assessed 12 months after the start of the study: the number of GPs and psychiatrists registered on the *med@psy* system, the number of patients managed *via* the system, the frequency of platform use and the average time between the GP’s referral and the psychiatric consultation provided through the *med@psy* system.

The fourth secondary objective is to characterize the population of patients who received an appointment for a psychiatric consultation within 48 hours *via* the *med@psy* system but who did not attend the scheduled consultation. Patients will be characterized according to the following criteria: age, sex, place of residence, psychiatric diagnosis and reason for referral by the GP.

### Statistics

All collected data will be summarized via descriptive statistics. Categorical variables will be described as frequencies and percentages, whereas continuous variables will be summarized using the median, minimum, maximum and mean (with standard deviation). Comparisons between groups will be conducted via chi² or Wilcoxon‒Mann‒Whitney tests, depending on the nature of the variable, to ensure comparability between groups. Statistical analyses can then be adjusted, and subgroup analyses can be carried out.

The main objective of this study was to compare the proportion of patients in each group who received outpatient follow-up at one month. A Farrington-Manning test will therefore be used to compare these proportions. The first secondary objective will involve a similar comparison of outpatient follow-up at three months between the two groups.

At one and three months, telephone interviews will be conducted by a psychologist to assess the Global Functioning Scale (GAF) score. The distribution of the GAF score between groups will be compared via a nonparametric Wilcoxon Mann‒Whitney test.

At 3 and 12 months, questionnaires will be administered to users of the *med@psy* system (GP and psychiatrists) to assess their overall satisfaction and report any concerns they may have.

System adherence will be assessed 12 months after the start of the study, on the basis of the number of GPs and psychiatrists registered on the platform, the number of patients treated *via* the system, the frequency of use, and the time elapsed between referral and the psychiatric consultation offered *via* the *med@psy* system.

Additionally, the characteristics (age, sex, pathology and reason for referral) of patients who obtained an appointment for a psychiatric consultation within 48 hours via the *med@psy* system but did not attend will be described. These nonincluded patients will have been informed of the research in advance and must not have expressed any opposition to their data being processed. The statistical significance threshold is 5%.

### Data

To collect the data required for the study, several databases will be consulted: the *med@med* digital platform, medical records of patients attending the CTS psychiatric emergency department, and data collected directly from patients during teleconsultations.

### Ethical considations

This protocol has been submitted to and accepted by the Ethics Committee (CPP Ile de France III – 2024-A01115-42). All research procedures were conducted in accordance with the principles outlined in the Declaration of Helsinki. The study has been registered on ClinicalTrials.gov under the identifier NCT06581874. Prior to participation, all patients received both oral and written information about the study and were included only if they did not express any objection to participation.

This study was conducted as a Category 3 research involving human participants (RIPH 3) under French law (Loi Jardé). For this type of study, informed consent is not required. Participants were provided with clear and appropriate information about the study objectives, the data collected, and their right to object to participation. Inclusion was based on the absence of objection. Investigator’s team assessed participants’ capacity to understand the information and express opposition at the time of inclusion; individuals unable to do so were not included. This information and non-opposition procedure was approved by the Ethics Committee [CPP Ile de France III] (approval number: 24.02552.000635).

### Status

Patient enrollment began on 22 January 2025. The first patient was included on March 3, 2025.

Inclusion will continue for 12 months, with final enrollment expected by 3 March 2026. Data collection and follow-up are scheduled to be completed by 10 June 2026. The results are expected in September 2026.

## Discussion

This study aimed to evaluate the implementation of outpatient follow-up after consultation with a psychiatrist via the *med@psy* system compared with follow-up after a psychiatric evaluation in the emergency department of the CTS. The *med@psy* system is a collaborative care system that enables GPs to schedule a psychiatrist consultation for their patients within 48 hours.

This study evaluated a new care pathway initiated by GPs, positioned as a potential alternative to emergency psychiatric consultation. It will be carried out under real-world conditions, taking into account existing difficulties and barriers to implementation. One potential risk identified is the possibility that GPs may not adopt or consistently use the *med@psy* system, which could affect the recruitment process and limit data collection.

The *med@psy* system was developed to address difficulties in accessing secondary mental health care, particularly for patients initially managed in primary care. Numerous barriers to the integration of mental health services into the primary care setting have been identified. This includes a low level of interest in mental health care provision among some GPs, increased workload and limited time, insufficient local mental health support, and limited resources for service delivery [12]. However, there are also enablers, such as the ability to understand the patient holistically and the convenience of service provision [12]. This new integrated care pathway via the *med@psy* system responds to facilitators while limiting obstacles. Referral to the platform is simple and takes less than 5 minutes. The *med@psy* system operates on a participatory and voluntary basis. This study highlights the crucial role of psychiatrists in primary care and provides information about the local mental health network. It is advisable to create and maintain up-to-date databases that provide GPs with information about psychiatrist availability, areas of expertise, and modes of practice, as this helps align referrals with appropriate psychiatric services [13]. Timely access to psychiatric consultations helps reduce frustration for both GPs and patients [13]. The *med@psy* system provides information about the various available referrals. Local educational interventions involving secondary care specialists and structured referral forms have also been shown to impact referral rates [14]. In addition, the *med@psy* system integrates reimbursement systems for unscheduled care, thereby enabling its use by GPs and psychiatrists. This approach helps to overcome financial barriers that might impede its implementation [15]. Taken together, these elements represent strong arguments in favor of the adoption and sustained use of the *med@psy* system by GPs.

This new care pathway benefits from being based on the existing network of healthcare practitioners while considering the realities of primary care practice. We also assume that this *med@psy* system goes beyond traditional interprofessional collaboration and constitutes a model of “integrated” care. The World Health Organization (WHO) defines integrated care as follows: *“The management and delivery of health services so that people benefit from a continuum of health promotion, disease prevention, diagnosis, treatment, disease management, rehabilitation and palliative care, across the different levels and sites of care in the health system, and according to their needs throughout life”* [16]. Other definitions have also been set out [17]. An essential aspect of integrated care is coordinating care effectively according to people’s needs [17]. It is therefore not just a question of collaboration but also of integrating new practices into primary care that considers the patient’s interests. Referring the patient directly to the psychiatric emergency department is a typical default pathway for the population and is sometimes not patient-centered.

This study has several limitations. It is subject to the capacity of the two pathways to take on the active files of patients. Factors such as regional medical demographics (general practitioners and psychiatrists) and the organizational structure of the CTS emergency department are beyond our control. In terms of patient profiles, the study did not focus on a single diagnostic category, thereby introducing clinical heterogeneity. The effectiveness of the *med@psy system* also relies on its appropriate use by GPs; excessive or indiscriminate referrals could risk overwhelming the system. Furthermore, the broader healthcare context including potential reforms or changes to reimbursement models may significantly influence care pathways but falls outside the scope of our control. Importantly, this study assesses only two specific types of care pathways, each with particular prerequisites. For example, the collaborative model requires patients to have an identified GP that excludes patients without a registered GP or those already engaged in psychiatrist outpatient care. Finally, the study did not evaluate patient adherence to follow-up or the clinical effectiveness of the outpatient care provided.

## Abbreviations

CTS: Toulon - La Seyne-sur-Mer Intercommunal Hospital Center
GPs: General practitioners
GAF: Global Assessment of Functioning
WHO: World Health Organization.
